# Sleep Telemedicine Practices: Checkpoint List and Practical Considerations in the COVID-19 Era

**DOI:** 10.3389/fneur.2021.664274

**Published:** 2021-04-20

**Authors:** Athanasios Voulgaris, Luigi Ferini-Strambi, Nicholas-Tiberio Economou, Evangelia Nena, Paschalis Steiropoulos

**Affiliations:** ^1^Medical School, Democritus University of Thrace, Alexandroupolis, Greece; ^2^Sleep Disorders Center, Department of Neurology, Scientific Institute Ospedale San Raffaele, Vita-Salute University, Milan, Italy

**Keywords:** COVID-19, obstructive sleep apnea, positive airway pressure, SARS-CoV-2, sleep telemedicine

## Background

The novel coronavirus disease (COVID-19) pandemic has forced clinicians to quickly adapt to this unprecedented situation and change, in many cases, their daily clinical practice and the management of their patients (1, 2). A practical approach to meet this need is the use of telemedicine (TM) in clinical activities, for instance in sleep medicine (3). Indeed, sleep TM provides a broad number of opportunities and can be utilized during the COVID-19 outbreak, promoting optimal health care and uninterrupted access to medical services for patients with sleep disorders (3). Sleep TM refers to remote exchange of sleep-related medical information using various communications aiming at improving patients' health (4). Sleep TM services can be offered either in a synchronous or asynchronous manner, principally formed in the following modalities: telediagnostics, teletherapy, telemonitoring, and teleconsultation (4). The American Academy of Sleep Medicine has recently published a position paper, in which an analysis of the terminology and the opportunities given by the sleep TM advancements are displayed (4). In addition, we recommend a comprehensive review on the topic (5).

Regarding the management of patients with obstructive sleep apnea (OSA) during the outbreak, sleep TM can reduce long waiting lists for patients, created due to the delays attributed to the necessary infection control and prevention practices. Moreover, several in-person medical visits that can be avoided [e.g., screening for obstructive sleep apnea (OSA), follow-up of already diagnosed patients, etc.] and performed remotely over sleep TM would provide a normal transition for sleep medicine practices during these stressful times (3).

In order to outline the necessary steps for sleep physicians regarding the practice of TM in the diagnosis and treatment of OSA patients and other sleep-related breathing disorders, we have created a simplified checkpoint list (Table 1).

**Table 1:** Approach to sleep telemedicine practices in patients with obstructive sleep apnea during the COVID-19 pandemic.

**Telediagnosis**	**Teletherapy**	**Telemonitoring**	**Teleconsultation**
✓ **Checkpoint 1—General medical information** 1) General medical information taken by the sleep physician in collaboration with the sleep technologist, including clinical history, family history, and optionally basic physical findings such as blood pressure and pulse rate where feasible 2) Demographic and anthropometric characteristics, comorbidities, upper airway surgeries, history of hospitalizations, and current medication use ✓ **Checkpoint 2—OSA screening** 1) Screening for symptoms related to OSA (witnessed apneas, snoring, daytime sleepiness, insomnia, gasping, morning headaches, fatigue, etc.) 2) Use of validated questionnaires for OSA (i.e., Berlin, Stop-Bang, NoSAS score) ✓ **Checkpoint 3—Home sleep apnea testing** 1) HSAT in adults with suspected OSA and uncomplicated medical history	✓ **Checkpoint 4—Informing the patient following the night of diagnostic sleep study** 1) Informing the patient, from the sleep physician, regarding the results of the sleep study and the possible treatment options ✓ **Checkpoint 5—Initiating positive airway pressure treatment or other treatment modalities** 1) Home PAP treatment (autotitrating) for patients with moderate to severe OSA 2) Download of the data from the PAP device and prescription of a PAP treatment device, accordingly 3) Discussion of the sleep physician with the patient for the various OSA treatment options beyond PAP especially for mild OSA (positional therapy, mandibular advancement device, upper airway surgery). Schedule one of them according to the patient's characteristics in selected patients or when PAP trial fails 4) Arrange follow-up visit according to the sleep center's practices (e.g., at 1 week, 3 months, 1 year)	✓ **Checkpoint 6—Follow-up of patients under PAP treatment** 1) Obtain patient's residual symptoms and adherence to PAP treatment by accessing the data from the PAP device online (e.g., data transfer from the manufacture's cloud) according to each sleep center's practices. Of note, the reliability of data download, although not optimal, it can be practical during the pandemic, especially in terms of hours of the device use 2) Troubleshooting of common issues that usually arise in the management of patients with OSA (renewal of prescription of PAP devices, mask leaks, discomfort, etc.) from the sleep technologist and sleep physician, or when alarms are set on by the provider's software (e.g., in case of low adherence to PAP use) 3) Regular assessment of other sleep and/or mood disorders, that may coexist with OSA (restless legs syndrome, insomnia) or might have been arisen or exacerbated due to the pandemic (i.e., anxiety, depression), with specific questionnaires and schedule for immediate management	✓ **Checkpoint 7—Teleconsultation** 1) General considerations to all patients during the pandemic: (a) sleep hygiene, (b) weight loss, (c) restrain from alcohol and tobacco use, (d) enhancement of physical activity, (e) proper OSA control, (f) avoid working up late especially for those who telecommute during the pandemic 2) Schedule of sleep clinic visits when necessary and obtain information regarding SARS-CoV-2-related symptoms 24 h upon arrival to the sleep center and according to the local hospital guidelines 3) Offer consultation in OSA patients who are diagnosed with COVID-19 and will require guidance regarding the treatment of their disease either at home or in-hospital, in collaboration with the caring physicians

*COVID-19, coronavirus disease 19; HSAT, home sleep apnea testing; OSA, obstructive sleep apnea; PAP, positive airway pressure; RLS, restless legs syndrome; SARS-CoV-2, severe acute respiratory syndrome coronavirus-2*.

## Discussion

In general, a stepwise approach, for the assessment of patients with suspected or previously diagnosed OSA, using sleep TM services and customized to each sleep center's practices, is suggested. In other words, evaluation of the interested individual should be divided into (a) the initial and (b) the follow-up visit. Finally, our perspective for adopting this algorithmic approach beyond the COVID-19 era is briefly discussed.

### Initial Evaluation

#### Checkpoint 1

First and foremost, general medical information should be the first step of the suggested checkpoint list. Specifically, in order to obtain medical history and record other related medical issues, all patients must be interviewed with a synchronous televisit, preferably via a clinical video telehealth (CVT) in order to have interactive communication and direct feedback from the patients.

#### Checkpoint 2

Screening for OSA symptoms will follow the general medical history taking. When necessary, and taken into consideration the patient's ability, the standardized medical history and all related material (e.g., screening questionnaire for OSA complemented with relevant information like OSA-related comorbidities) can be downloaded and self-answered by the interested individual from the sleep center's website. Thereafter, patients' medical documents can be transferred electronically (e.g., email, upload) to the sleep center account and be saved in an electronic patient file using the electronical database of each hospital. The sleep physician can use this, during the online medical examination/interview, in order to speed up the process.

#### Checkpoint 3

OSA diagnosis with a sleep study is recommended, as it is the most important part of the present algorithm. When clinical suspicion for OSA is high, home sleep apnea testing (HSAT) is a valid option for uncomplicated OSA (6). The sleep center, staff should be responsible for dispensing its HSAT device along with instructions depicted in a standard brochure on how to wear it, start its use, and end up its recording. This would allow knowledge of the subjects that have recently used the HSAT device ensuring appropriate time intervals for effective disinfection.

#### Checkpoint 4

In the context of OSA diagnosis, an interactive televisit will inform the patient for the results of the sleep study and the various treatment options. After completing this virtual visit, the results can be electronically communicated to patients via an email. Additional video links and supplementary material for the treatment options can be also sent.

#### Checkpoint 5

OSA treatment is guided according to the guidelines of the American Academy of Sleep Medicine, which can be practically divided into treatment with positive airway pressure (PAP) and other non-PAP therapy options (7). Patients with moderate and severe OSA or patients with mild OSA and symptoms like excessive daytime sleepiness and/or associated comorbidities (i.e., hypertension) will normally require treatment with PAP. In these cases, and in the context of sleep TM, an autotitrating PAP trial for home OSA treatment can be applied, and subsequently, the clinicians can prescribe PAP treatment devices, according to the home PAP study results. Before PAP titration, another videoconference is also necessary to explain the PAP trial procedure, answer to patients' questions, and offer live instructions on how to customize the device and mask, according to the patient's requirements and preferences.

Another CVT, following the night of the therapeutic trial and after accessing the PAP device data, should inform the patient regarding the results of this trial and their device use and related information, as well as inform on various technical parameters on the PAP device. Although several barriers at these important steps would normally arise, a practical approach to overcome them is to perform a live demonstration of both the HSAT and the PAP trial by the sleep technologist and under the supervision of the sleep physician. In case where the PAP trial fails, when the patient cannot tolerate the PAP device, or the individual has mild OSA; alternative, non-PAP therapy options should be offered. For instance, referral for mandibular advancement device or initiation of positional therapy and schedule follow-up visits according to the sleep center's routine are alternative treatment suggestions (at 1 week, 3 months, 1 year, etc.).

### Follow-Up Visit

#### Checkpoint 6

One of the key elements of OSA management is to obtain regular follow-up of OSA patients. Special attention with telemonitoring should be offered to PAP-treated patients in order to optimize their PAP adherence (8). Follow-up of patients under PAP treatment is usually standardized according to each sleep center's practices. Nevertheless, unplanned visits can be immediately arranged in the context of low adherence to PAP therapy when automatic alarms by the provider software are set on. Advanced wireless models of the novel PAP devices, which include cloud-based platforms and aid in improved PAP adherence and efficacy monitoring, can be helpful (5, 9). Additionally, several educational TM tools can also lead in improved patient–provider interaction and result in efficient patient's adherence to PAP without the need for face-to-face consultations (9, 10). Moreover, general issues can also be addressed via routine televisits, such as technical demands (i.e., PAP prescription renewals), patient's overall health status, or other sleep and mood issues that might have emerged (e.g., insomnia, anxiety).

#### Checkpoint 7

The sleep centers are often called to provide consultation on matters that are addressed beyond OSA diagnosis and treatment. Recommendations for general health and sleep hygiene should be offered to all patients. In addition, clarifications with written instructions should be sent to patients when in-person visit to the sleep center is necessary in order to simplify the procedure and to shorten their visit time.

Moreover, sleep centers should be prompted to provide specific guidance with the various e-communications for their OSA patients, who are diagnosed with COVID-19, and stay updated with the current recommendations regarding medical services during the pandemic. Specific recommendations are outlined elsewhere (1, 11) and briefly include self-isolation and continued use of their PAP treatment, preferably a non-vented mask, placement of exhalation filter, and avoidance of humidifier, to minimize the risk of PAP-induced droplet transmission.

Certainly, the present checkpoint list regarding sleep TM practices has limitations, as it is addressed mainly to patients who have internet access and who can communicate with the sleep center over modern applications. Nevertheless, older patients who are unable to be assessed with sleep TM can still benefit from the sleep TM options if, for instance, they have younger relatives who can arrange for them all the necessary procedures. During the pandemic, this is especially important, as this is the population at a higher risk for poor COVID-19 outcomes if they get infected with the coronavirus. Any remote medical assessment minimizes the risk of infection.

In summary, two important considerations should be looked into at each of the abovementioned steps when practicing sleep TM during the COVID-19 pandemic. The first refers to the specific regulations, either at regional or national level, that may limit this approach (e.g., data protection and storage, commercial terms, and regulations, etc.); the second consideration refers to the sleep centers, which should always prioritize infection control strategies in order to lessen the transmission of severe acute respiratory syndrome coronavirus 2 (SARS-CoV-2) to both patients and staff of the sleep center.

### Perspective of Sleep Telemedicine Beyond the COVID-19 Era

Sleep TM can be proven particularly useful during the pandemic and can also serve as a normal transition of sleep medicine practices between the outbreaks of the coronavirus (1). Effectively adapting this simple checkpoint list would lead to continuous management of patients with OSA, minimizing the risk of transmission and providing the best available care for these patients (1). In addition, sleep centers without having previous experience of practicing sleep TM can easily apply this algorithm and extend these practices beyond the COVID-19 era. More importantly, a fully equipped Virtual Sleep Center, run by sleep specialists and in collaboration with primary care physicians, would overcome several barriers that traditionally arise in the routine of a sleep center such as long waiting lists, enable quick access to sleep medicine in case of remote areas, and solve problems of PAP adherence, titration settings, and mask discomfort (12). Finally, this perspective could have significant economic benefits (13).

In conclusion, telemedicine is an easily applied method that helps to overcome barriers that have arisen in sleep medicine practice due to the COVID-19 pandemic. A structured checkpoint list can simplify and enhance the effective management of OSA patients. Nonetheless, these novel approaches cannot replace in-person contact between the patient and the physician, and when deemed necessary, in-laboratory testing should be prioritized over remote sleep assessment.

## Author Contributions

All authors participated in writing, revising, and approving the final draft of the manuscript.

## Conflict of Interest

The authors declare that the research was conducted in the absence of any commercial or financial relationships that could be construed as a potential conflict of interest.
